# PAFc, a Key Player in MLL-rearranged Leukemogenesis

**DOI:** 10.18632/oncotarget.181

**Published:** 2010-10-05

**Authors:** Jiaying Tan, Andrew G. Muntean, Jay L. Hess

**Affiliations:** Department of Pathology, University of Michigan Medical School, Ann Arbor MI, USA, 48109

**Keywords:** oncoprotein, leukemia, oncotarget, transcription

## Abstract

Recent studies identified an interaction between the Polymerase Associated Factor complex (PAFc) and Mixed Lineage Leukemia (MLL), including MLLrearranged oncoproteins. This interaction is critical for MLL transcriptional activity and MLL-rearranged leukemogenesis. Here, we discuss the potential molecular role of the PAFc in transcriptional dysregulation of MLL target genes and the interplay between PAFc and MLL-rearranged oncoproteins in leukemogenesis.

## INTRODUCTION

**Figure 1:**
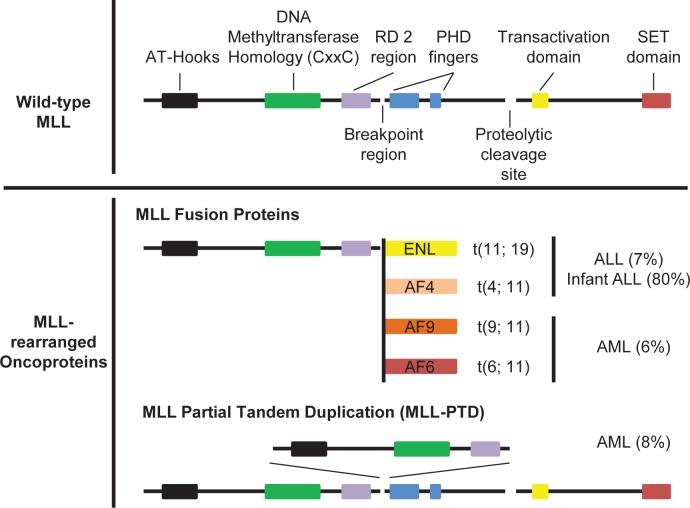
Schematic of wild-type MLL and MLL-rearranged oncoproteins Major functional domains and the proteolytic cleavage site of wild-type MLL are indicated. MLL fusion proteins consist of the N-terminus of wild-type MLL (up to the breakpoint region) fused in frame with a translocation partner (either a nuclear protein, such as ENL, AF4 and AF9, or a cytoplasmic protein, such as AF6). MLL-PTD is generated by exon duplication of the sequences encoding the N-terminus of wild-type MLL at the breakpoint region [32].

The mixed lineage leukemia gene *MLL*, the human homolog of the *Drosophila* trithorax gene, encodes a histone H3 lysine 4 (H3K4) methyltransferase that positively regulates multiple homeobox transcription factors, including *Hoxa9* and *MEIS1*, which are pivotal for leukemogenesis [1]. *MLL* rearrangements that generate MLL-rearranged oncoproteins are associated with a variety of acute lymphoid and myeloid leukemias that have a dismal prognosis [2]. To date, more than 50 different translocation fusion partners have been identified, among which the most common are nuclear proteins with transcriptional activating activity [2]. In acute lymphoblastic leukemias (ALL), the most common translocations are t(11;19) and t(4;11), resulting in the fusion proteins MLL-ENL and MLL-AF4, respectively. In contrast, the t(9;11) translocation, resulting in the MLL-AF9 fusion protein, is more frequently found in acute myeloid leukemias (AML). In addition to the nuclear translocation partners, another class of MLL fusion partners consists of cytoplasmic proteins that contain dimerization domains, such as AF6. Dimerization of these MLL fusion proteins leads to potent transcriptional activation and is essential for their leukemogenic capacity; however, the detailed leukemogenic mechanism remains elusive [3, 4]. *MLL*-related translocations are also commonly observed in secondary acute leukemias after topoisomerase inhibitor treatment [5]. In addition, around 8% of AML patients with normal cytogenetics harbor internal tandem duplications of partial MLL N-terminal sequence, known as *MLL-PTD* (Fig. 1). Overall, genetic lesions in the *MLL* gene are associated with more than 80% infant leukemias and approximately 10% adult leukemias [2].

MLL is a ubiquitously expressed multi-domain protein required that has been shown to be essential for the survival of hematopoietic stem and progenitor cell populations [2]. Although multiple featured domains are present throughout the wild-type MLL protein, only the N-terminus containing the Menin interaction domain, AT-hooks and CxxC-RD2 domain (up to the break point region) is invariably retained in all *MLL*-rearranged oncoproteins, whereas the Plant Homeodomain (PHD) and the SET domain, which is required for the histone methyltransferase activity, are consistently deleted (Fig. 1) [6]. Menin, a tumor suppressor encoded by the *MEN1* gene, has been shown to directly interact with the extreme N-terminus of MLL, and this interaction is essential for MLL-rearranged leukemogenesis [6, 7]. A previous study has demonstrated that this interaction also involves a chromatin-associated protein, LEDGF (lens epithelium-derived growth factor) [8]. The CxxC domain selectively binds unmethylated CpG DNA sequence and aids in the localization of MLL fusion proteins to the target loci, protecting the corresponding regions against DNA methylation [9]. However, the role of the CxxC-RD2 region, particularly the RD2 region immediately N terminal to the breakpoint region, in the cellular activities of wild-type MLL or MLL fusion proteins remains elusive. The importance of this region is highlighted by recent work by Bach et al. who clearly demonstrated that the DNA-binding affinity alone does not fully account for the indispensible role of this region in leukemogenesis, indicating the presence of uncharacterized activities/interactions critical for MLL-rearranged leukemogenesis [10].

Our recent study, as well as the work from Milne *et al*., helps to clarify the role of the CxxC-RD2 region in MLL-rearranged leukemogenesis. Using mass spectrometry, we found that the Polymerase Associated Factor complex (PAFc) interacts with this region and that this interaction is critical for MLL transcriptional activity as well as leukemogenesis [11, 12]. PAFc is a multi-protein complex, with the core components of PAF1, LEO1, CDC73, CTR9 and WDR61 [13-16]. Increasing evidence has revealed that PAFc plays important roles in a wide range of biological processes, including the initiation, elongation and termination of gene transcription, cell cycle regulation, mRNA processing, H2B monoubiquitination, H3K4 methylation and H3K79 methylation [17-19]. In addition, several components of PAFc are known to play important roles in cancer biology. For instance, PAF1 is shown to be upregulated or amplified in prostate cancer, whereas CDC73 has been associated with multiple types of human cancers, such as breast, renal and gastric cancer, as well as the hyperparathyroidism-jaw tumor syndrome [16, 17]. Previous studies have demonstrated that the yeast PAF complex is required for the recruitment of the yeast Set1 methyltransferase complex, termed COMPASS, to RNA polymerase II; the interaction is also indispensible for both COMPASS mediated histone H3K4 and Dot1L mediated H3K79 methylation [14, 20]. Given these results, it seemed to be likely that the MLL complex, the human homolog of COMPASS, is also physiologically and functionally associated with PAFc. Indeed, by mass spectrum analysis, we demonstrated that PAFc interacts with the CxxC-RD2 region of MLL, a region that is always retained in MLL-rearranged oncoproteins. Detailed mapping revealed two interaction sites flanking the CxxC domain with two individual components of PAFc. Most importantly, we were able to show that the PAFc-MLL interaction enhances the transcriptional activation by MLL-AF9 and plays an indispensible role in MLL-AF9 mediated transformation [11].

Characterization of the PAFc-MLL interaction provides valuable insight into the mechanisms of *MLL*-rearranged leukemogenesis. The best defined target genes of MLL are the clustered homeobox (*Hox*) genes, a transcription factor family important in cell fate determination during development. Among these targets, Hoxa9 and its cofactor Meis1 have been shown to be crucial for MLL-rearranged leukemogenesis. Normally, *Hoxa9* and *Meis1* are only briefly expressed in hematopoietic stem cell and progenitor cell populations and are then rapidly down regulated during hematopoietic differentiation [21-23]. However, in the presence of MLL-rearranged oncoproteins, both remain expressed at high levels, which accounts for their leukemogenic capacity. Although many interaction partners of MLL-rearranged proteins have been identified and shown to be important in leukemogenesis, it remains unclear what are the exact molecular mechanisms responsible for the dysregulation of the expression of these target genes.

**Figure 2:**
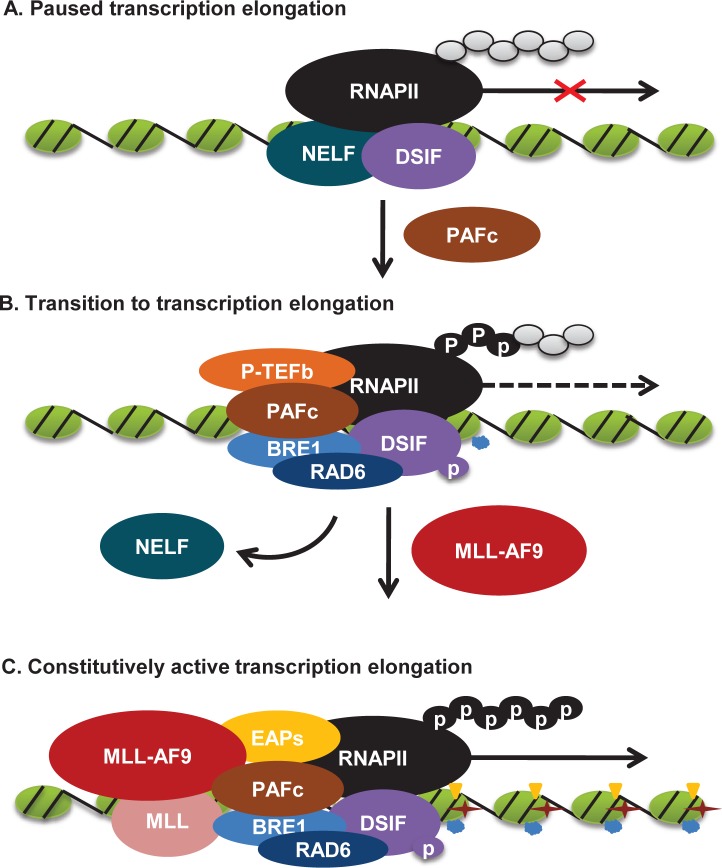
Schematic of a potential mechanism of MLL-rearranged leukemogenesis (A) In the absence of PAFc, RNA polymerase II (RNAPII) elongation is inhibited by the negative elongation factor (NELF) in collaboration with the DRB sensitivity-inducing factor DSIF. (B) DSIF recruits PAFc that directly interacts with the E1/E2 ubiquitin ligase complex BRE1/RAD6, resulting in histone H2B monoubiquitination. Recruitment of positive transcription elongation factor b (P-TEFb) blocks the negative actions of NELF and DSIF by P-TEFb-dependent phosphorylation of RNAP II CTD and DSIF, priming the target promoter for transcription elongation (C) The interaction between PAFc and the most common MLL-rearranged oncoproteins (represented by MLL-AF9) recruits the ENL-associated proteins (EAPs) that include multiple common MLL translocation partners, DOT1L and P-TEFb to the target loci, promoting H2B monoubiquitination (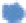), H3K4 methylation (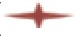) and H3K79 methylation (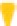), resulting in constitutively activated transcription.

Aside from our identification of the PAFc-MLL interaction, several lines of evidence also indicate the direct role of PAFc in *Hox* gene dysregulation in MLL-rearranged leukemias. First, increasing evidence suggests that a significant mechanism for *Hox* gene expression is mediated through regulating transcriptional elongation [24], a process in which PAFc has been known to play a key regulatory role [16]. Second, we observed a significant dose-dependent transcriptional activation of the *Hoxa9* promoter induced by MLL-AF9 overexpression, whereas wild-type MLL overexpression only delivered a somewhat more muted response, suggesting the differential roles of PAFc in cellular activities of MLL-rearranged oncoproteins vs. wild-type MLL. Third, a previous study by Chen et al. has demonstrated that the susceptibility of hematopoietic progenitors to MLL-AF9-induced transformation decreases along differentiation [25], consistent with the decreasing PAFc expression along hematopoietic differentiation shown by our work and others [11, 26]. Based on these findings, a potential mechanism for MLL-rearranged leukemogenesis is that by interacting with PAFc, MLL-rearranged oncoproteins are able to stably engage the basic transcription elongation machinery at target loci, such as *Hoxa9* and *Meis1*, to constitutively activate transcription, leading to leukemogenesis. Therefore, PAFc may be a crucial component mediating the dysregulation of normal transcription elongation of MLL target genes by MLL-rearranged oncoproteins (Fig. 2).

Under normal conditions, *Hoxa9* is expressed in primitive hematopoietic cells, playing a significant role in early hematopoiesis [27, 28]. During hematopoietic differentiation, *Hoxa9* expression is rapidly silenced, likely by pausing transcription elongation, an important mechanism regulating *hox* gene expression in *Drosophila* [24, 29]. In this case, although RNA polymerase II (RNAPII) still localizes at the promoter region, its C-terminal domain (CTD) is unphosphorylated, and transcription elongation is inhibited by the negative elongation factor (NELF) in collaboration with the DRB sensitivity-inducing factor (DSIF) (Fig. 2A). DSIF recruits PAFc that directly interacts with the E1/E2 ubiquitin ligase complex BRE1/RAD6, resulting in histone H2B monoubiquitination. Meanwhile, the recruitment of positive transcription elongation factor b (P-TEFb) reverts the negative actions of NELF and DSIF by P-TEFb-dependent phosphorylation of RNAP II CTD and DSIF [16]. Thus, in the presence of PAFc, the target gene promoter region can progress to the active elongation stage (Fig. 2B). It is worth noting that this status is probably a temporary transition stage, dynamically regulated by cell-specific mechanisms, such as the abundance of PAFc, the binding affinity of other transcription elongation machinery components determined by the phosphorylation level of RNAP II CTD, the recruitment of histone methyltransferases, such as wild-type MLL and DOT1L, exerting H3K4 and H3K79 methylation, respectively, and the regulation of their enzymatic activities. For instance, in hematopoietic stem cells and early-stage progenitor cells, PAFc is expressed at a high level; therefore, this temporary transition status is more likely to progress into a fully active elongation stage, which in turn leads to the *Hox* gene expression. In contrast, in the differentiated cells, PAFc downregulation may revert this transition status back to the inactive transcription stage, silencing the *Hox* gene expression. The dynamics of the multiple regulatory mechanisms is likely to be disrupted by MLL-rearranged oncoproteins, such as MLL-AF9.

The most common MLL-rearranged oncoproteins, including MLL-AF9, MLL-ENL and MLL-AF4, are known to interact with a protein complex termed ENL-associated proteins (EAP) or a closely related complex called AEP for AF4 family/ENL family/P-TEFb complex [11]. By interacting with PAFc, these MLL-rearranged oncoproteins recruits EAP that includes DOT1L, P-TEFb and multiple common MLL translocation partners to the target loci, promoting H3K79 methylation, resulting in dysregulated constitutively active gene expression (Fig. 2C). In addition, wild-type MLL has recently been shown to synergize with MLL-AF9, which furthering increases the H3K4 methylation level, and presumably contributing to target gene transcription [30]. Notably, in the study by Chen *et al*., the authors showed that LSK (Lin^-^Sca1^+^C-kit^+^) stem cells, but not the more differentiated committed granulocyte-monocyte progenitors (GMPs), can be transformed by MLL-AF9 under endogenous regulatory control, suggesting that under physiological conditions, additional linage-specific transcription factor(s) or coactivators(s), other than the MLL-rearranged oncoproteins are critical for leukemogenesis [25]. Given the downregulation of PAFc during hematopoietic differentiation, it is possible that PAFc at least partially accounts for the susceptibility of different progenitor populations to MLL fusion protein induced leukemogenesis.

A number of questions remain regarding the mechanism of the PAFc-MLL interaction in MLL-rearranged leukemogenesis. First, apart from the most common *MLL* translocations resulting in MLL fusion proteins with a nuclear translocation partner, MLL fusion proteins with cytoplasmic partners and MLL-PTD have not been extensively studied. Therefore, the leukemogenic mechanisms of MLL-PTD and MLL-rearranged oncoproteins with a cytoplasmic partner, both of which in effect involve duplication of the N-terminus of MLL (up to the breakpoint region) by either intramolecular partial tandem duplication or intermolecular dimerization, are unknown [31]. Given the pivotal role of PAFc in MLL-rearranged leukemogenesis, it will be important to determine if either of these two types of MLL-rearranged oncoproteins involves enhanced physical or functional interaction with PAFc. Second, it is still unclear how, and to what extent, PAFc plays differential roles in the cellular activities of MLL-rearranged oncoproteins vs. wild-type MLL. It will be important to determine if such a therapeutic window exists for targeting PAFc, for example, through targeting MLL-PAFc interaction with small molecule inhibitors, which could be used as a new therapy for MLL-rearranged leukemias.
